# Plant-Soil-Microbiota Combination for the Removal of Total Petroleum Hydrocarbons (TPH): An In-Field Experiment

**DOI:** 10.3389/fmicb.2020.621581

**Published:** 2021-01-26

**Authors:** Daniela Zuzolo, Carmine Guarino, Maria Tartaglia, Rosaria Sciarrillo

**Affiliations:** Department of Science and Technology, University of Sannio, Benevento, Italy

**Keywords:** sustainable remediation, Poaceae, Fabaceae, total petroleum hydrocarbons, mycorrhizae, plants

## Abstract

The contamination of soil with total petroleum hydrocarbons (TPH) may result in dramatic consequences and needs great attention, as soil rehabilitation would need more effort from a sustainability perspective. However, there is still no known general method since the remediation technology is strictly site-specific. Adaptive biological system dynamics can play a key role in understanding and addressing the potential of situ-specific biological combinations for soil pollutants removal. The potential worst-case of TPH contamination reflects soil affected by heavy industrial activities, such as oil refineries. Therefore, the experimental trial was conducted on a 2,000 m^2^ area from a contaminated site located in northern Italy. We evaluated the remediation potential over time (270 days) assessing (i) the phytoremediation efficiency of two species of Poaceae (*Festuca arundinacea* Schreb. and *Dactylis glomerata* L.) and two species of Fabaceae (*Medicago sativa* L. and *Lotus corniculatus* L.) and (ii) the role of the indigenous bacteria flora and endo-mycorrhizae consortium addition in plant growth promotion. We also induced resistance to contamination stress in a field experiment. Thirty-three indigenous bacteria selected from the contaminated soils showed marked plant growth promotion. Moreover, functional metagenomics confirmed the metabolic capability of hydrocarbon-degrading microorganisms living in the polluted soil. Our data showed that soil enzymatic activities increased with hydrocarbon degradation rate after 60 days. Both Poaceae and Fabaceae resulted in remarkable remediation potential. Stress markers and antioxidant activity indicated that the selected plant species generally need some time to adapt to TPH stress. In conclusion, our evaluation implied both the rhizosphere effects and functional features of the plant and suggested that plants should (i) have marked tolerance to specific contaminants, (ii) be characterized by an extensive root system, and (iii) be susceptible to arbuscular mycorrhizal fungi (AMF) infection.

## Introduction

The soil pollution produced by total petroleum hydrocarbons (TPH), as aliphatic, aromatic, heterocyclic, and asphaltene/tar hydrocarbons are together termed, constitutes a serious issue worldwide due to eco-toxicity caused by their strong persistence in the environment (Hussain et al., 2019). Most of this pollution has resulted from oil exploration, transport, and refining as well as via the lack of waste oil recycling and the disposal of hazardous oil wastes into landfill areas without sufficient management (Khudur et al., 2019; Grifoni et al., 2020). All these anthropic activities have further increased the number of contaminated sites (Koshlaf and Ball, 2017). The consequences of this pollution can be dramatic for soil ecological function since it can lead to a drastic reduction of the soil microbial biomass or the stunted growth of plants (Hussain et al., 2018). Among TPH, long-chain aliphatic hydrocarbons are a group of very recalcitrant compounds that reduce the availability of nutrients in soils, significantly increase water content, and immobilize hydrophobic complexes, thus indirectly affecting the soil’s ecological role (Devatha et al., 2019). Soil pollution also affects food security, both by reducing the agricultural production of the soil (as a consequence of plant metabolism impairing and microbial biomass harming) and by making crops unsafe for consumption (Kiamarsi et al., 2020). Hence, detoxification of TPH-contaminated soils has long been a worldwide challenge and has been addressed in various ways in recent years. Therefore, physical-chemical methods have been widely used and are still applied, including excavation, desorption, thermal destruction, soil washing, soil flushing, stabilization, soil vapor extraction, and many others (Vidonish et al., 2016; Ossai et al., 2020). These traditional methods have the advantage of solving the problem in a short amount of time, but costs could be high and the environmental matrices are usually compromised. However, a group of biological technologies capable of degrading hydrocarbons is currently emerging (Guarino and Sciarrillo, 2017; Wang et al., 2019; Ossai et al., 2020). The key benefits are that they act *in situ*, are low cost, and have a low environmental impact (Palmroth et al., 2006; Gaur et al., 2018). There are different biological approaches to the remediation of TPH contamination, such as bio-pile, biostimulation, bioaugmentation, and phytoremediation, all with a single final objective: the complete mineralization of hydrocarbons (Guarino et al., 2017). Lately, numerous laboratory scale studies have clarified in different ways the potential of these technologies in well-controlled environments but field applications are still not widespread and are considered less attractive than physic-chemical methods (Hoang et al., 2021). Currently, the rising understanding of soil biology and of the natural partnerships existing between the various living organisms occurring in soil (both plants and microbiota) is leading to a growing interest in the application possibilities of adaptive biological dynamics in remediation (Burges et al., 2017; Guarino et al., 2017). However, a greater effort to study these soil dynamics, which primarily affect the rhizosphere, is required to meet the challenge of soil rehabilitation. Generally, the rhizosphere zone has a strong physical and bio-chemical activities network influencing degradation mechanisms of xenobiotics such as hydrocarbons. Degrading bacteria, growth promoters, and ecto- and endophytic fungi are the protagonists (together with plants and, in particular, roots) of interesting degradation co-metabolic processes (Pawlik et al., 2017). In this context, plants play a key role as they shape the conditions for a specific microbial colonization through root exudation (Guyonnet et al., 2018). In addition, root colonizing symbiotic microorganisms, such as arbuscular mycorrhizal fungi (AMF), can play a crucial role in (i) improving the growth stages of the plant, (ii) increasing the biodegradation activity in the rhizosphere, and (iii) enhancing hydrocarbons’ uptake at the root level (Rajtor and Piotrowska-Seget, 2016).

The rhizosphere effect is therefore of primary importance in soil reclamation from TPH contamination and is based on mutual benefits brought about by the various organisms through the occurrence of roots exudates, bacterial degrading enzymes, production of growth promoters. and many other molecules which play an important role in regulating the soil bioactivity (Pawlik et al., 2017). It is also necessary to correctly associate this strong potential with the opportunities offered by preparing the land in the best conditions to receive these biological complexes (Liu et al., 2015). In fact, biological systems integrated with suitable agronomic practices could be the keystone for future biological remediation of organic xenobiotic species. Most phytoremediation studies have focused on industrial contaminated soils, which represent the worst-case scenario. In these contexts, a complex approach simultaneously involving agricultural practices and integrated biological systems appears as a suitable strategy to both decrease pollutant concentrations and restore agricultural soil functions over time. However, *in situ* research of phytoremediation at the metaorganism level (hosting plant and associated microbiome) has long been underestimated and most current studies in the field of hydrocarbons remediation have targeted only one specific group of TPH (i.e., PAHs) (Thijs et al., 2016; Hoang et al., 2021). With this study, we aimed at extending a laboratory-scale experiment (Guarino et al., 2017), in which an evaluation of three bioremediation approaches (natural attenuation, biostimulation, and bioaugmentation) of a TPH-contaminated soil was conducted, to the real-scale. In this in-field experimental study, the remediation of TPH-impacted soils has been evaluated by testing the interaction of four plant species (*Festuca arundinacea*, *Dactilys glomerata*, *Lotus corniculatus*, and *Medicago sativa*), with indigenous bacteria and inoculated mycorrhizal fungi in the rhizosphere. Our study aimed to (i) examine the TPH rhizodegradation suitability of the experimented plant-microbiome partnership, (ii) determine bacterial diversity and how our treatment would shift it, (iii) assess the potential benefit of synergistic cooperation between the metaorganism in the presence of abiotic stresses (soil pollution), and (iv) propose a method well-suited to field experimentation.

## Materials and Methods

### Experimental Design

The in-field experimental trial was conducted on a 2,000-m^2^ area from a decommissioned refinery in the north of Italy (Supplementary Figure S1) characterized by a history of pollution by petroleum hydrocarbons (predominantly diesel). The area was composed of four experimental subzones (named A, B, C, and D) of about 500 m^2^ each.

The experimental design included the use in all subzones of optimum agronomic practices (landfarming and fertilization) and selected plant species (phytoremediation). Mycorrhizae were employed to guarantee plant development promotion as well as beneficial outcomes linked to contamination stress resistance. Landfarming was performed at the beginning of the experiment in all four subzones by the turning of the soil up to a depth of 1.20 m. This operation was carried out three times at regular intervals with the aid of an excavator. The time interval (weekly) between one repetition and another of the intervention was performed to favor better aeration of the substrates, allowing the reconstitution of the microflora in a pervasive manner (United States Environmental Protection Agency [USEPA], 2017).

Fertilization with the mixture of NPK (105 kg/ha of N, 85 kg/ha of P, and 85 kg/ha of K) was carried out with the aforementioned soil turning interventions to ensure a good supply of macronutrients for plants and edaphic microflora.

Irrigation water, which conformed to the Italian legislation (Legislative Decree, 152/2006, data not shown), was supplied using an automatic drip irrigation system (3 liters per square meter). In all subzones, the bioaugmentation process made use of a chosen rhizosphere microorganism mixture (15 g for m^2^) composed of fungal endomycorrhizae (*Acaulospora colombiana*, *Rhizophagus clarus*, *Rhizophagus intraradices, Rhizophagus irregularis Claroideoglomus etunicatum, Funneliformis mosseae, and Funneliformis geosporum*) to investigate partnering of mycorrhizal fungi with native bacteria. *Festuca arundinacea*, *Dactilys glomerata*, *Lotus corniculatus*, and *Medicago sativa* plant species were chosen for soil phytoremediation since the efficiency of Fabaceae and Poaceae in both stimulating the rhizodegradation process and accumulating the hydrocarbons has been recognized by scientific literature (Gao and Zhu, 2004; Hall et al., 2011). *Festuca arundinacea* seeds (40 g/m^2^) were planted in subzone A, *Dactilys glomerata* (40 g/m^2^) in subzone B, *Lotus corniculatus* (57 g/m^2^) in subzone C, and *Medicago sativa* (57 g/m^2^) in subzone D. A neighboring uncontaminated area (CPH) was used as control.

To assess the advancement of the remediation process, soil samples were gathered prior to the experimental trial (BPH) and after the experimental trial (APH) at different periods: days 0 (pretrial-Control, January 2017, T_0_), 90 (March 2017, T_1_), 150 (May 2017, T_2_), 210 (July 2017, T_3_), and 270 (September 2017, T_*f*_). The soil samples were collected at a 0.40 cm depth in glass jars, labeled, and stored at 4°C for further analysis.

To evaluate the effects of plant stress, in each sub-area, thirty-five plant samples (separated in roots and leaves) were collected as indicated (black dots) in Supplementary Figure S1. The roots and leaves of each plant were collected, stored in labeled jars at approximately 4°C, avoiding cross contamination, and transported to the laboratory until analyzed. All samples were analyzed in triplicate.

Metagenomic survey for the analysis of microbial community composition and function of soil included the sampling of (i) contaminated soil prior to the experimental trial (BPH), (ii) after the experimental trial (APH), and (iii) on soil of neighboring uncontaminated area (CPH) used as control. Each sample was composite (as recommended by Vestergaard et al., 2017 and references therein) and represented four randomly selected sampling locations within the indicated areas. Soil samples were immediately placed on ice in a cooler and transported to the laboratory. A half of each sample was frozen in liquid nitrogen and stored at −80°C for subsequent molecular analysis. The remaining half was air-dried and then sieved through a 2-mm-pore-size sieve and finally stored at 4°C for further analysis. Along with metagenomics, the physical-chemical assessment of soil samples from control as well as contaminated soils (both before and after the treatment) were carried out (following recommendations of Vestergaard et al., 2017). All samples were analyzed in triplicate.

### Analyses

#### Isolation of the Autochthonous Soil Bacteria

A quantity of 1 g of homogenized and sieved rhizosphere soil of the contaminated chosen area prior (BPH) to the experimental design was suspended in Erlenmeyer flasks (in duplicate) containing 50 ml of a mineral media at pH 6.8 with the addition of 5% crude oil as a carbon source. Following the method described in Guarino et al. (2017), cultures were transferred in Erlenmeyer flasks containing 250 ml of a mineral media and incubated in an orbital shaker at 28 °C and 200 rpm for 5 days. Subsequently, in order to isolate the maximum number of strains and avoid the overlooking of slower growing colonies, 100 ml of serial 10-fold dilutions of bacterial cultures were spread on two different solid media: LB and R2A (Sigma-Aldrich, Milan Italy). After 9 days of incubation at 28°C, a total count of colonies was done. Fifty-one colonies per media had been achieved and preserved and they were randomly selected and maintained as pure cultures per samples.

#### Molecular Analysis of Autochthonous Bacteria

The molecular identification of the autochthonous bacteria present in the contaminated soil prior (BPH) and after (APH) the experimental design was obtained following the method reported in Guarino et al. (2019). Two grams of soil were used for the isolation of genomic DNA using the PowerSoil DNA Isolation Kit (MoBio Laboratories Inc., United States), which is effective at removing PCR inhibitors from even the most difficult soil types, according to the producer’s protocol. The isolated DNA were quantified by Nanodrop 1000 Spectrophotometer. 16S rRNA gene amplification was performed by using the eubacterial-specific primers (F27a: AGAGTTTGATCCTGGCTCAG; R14 92a: GGTTACCTTGTTACGACTT). Polymerase chain reaction (PCR) were conducted with GoTaq^®^ Polymerase (Promega) according to the manufacturer’s instructions. PCR-amplified DNA was sequenced with a BigDye^®^ Terminator v3.1 Cycle Sequencing Kit (Applied Biosystems Inc., United States) adopting an automated DNA sequencer (ABI model 3500 Genetic Analyzer). Nucleotide sequences were edited and assembled with Lasergene version 11.2.1 (DNASTAR^®^) and The BLAST search server^1^ was used to search for nucleotide sequence homology of the 16S region for bacteria.

#### *In vitro* Assessment for Plant Growth Promoting (PGP) Activities

The isolated bacteria from BPH soil samples were tested individually for PGP activities to depict bacteria features which might indirectly enhance plant growth. Indolacetic acid (IAA) production, siderophores release, exopolysaccharides (EPSs), and ammonia production were screened following the method of Guarino et al. (2017).

#### Library Preparation and Sequencing TPH Degrading Genes

Library preparation and sequencing were performed in soil of the contaminated chosen area prior (BPH) and after (APH) the experimental trial and in soil of a neighboring uncontaminated area (CPH) used as control. Analysis were centered on the genes engaged in hydrocarbon degradation research (Guarino et al., 2020).

The Ovation^®^ Ultralow V2 DNA-Seq kit (Nugen, San Carlos, CA) was adopted for producing libraries. The samples were quantified and quality tested using the Qubit 2.0 Fluorometer (Invitrogen, Carlsbad, CA) and Agilent 2100 Bioanalyzer (Agilent Technologies, Santa Clara, CA). The sequencing run was performed on MiSeq (Illumina, San Diego, CA), sequencing 300 cycles per read. Base calling and demultiplexing were performed on integrated analysis software (Guarino et al., 2019, 2020). The sequenced genes, obtained from Uniprot, are listed below: Alkane-1 monooxygenase (Uniprot code O05895 and P12691), Cytochrome P450 alkane hydroxylase (Uniprot code A9CMS7 and Q2MHE2), Protocatechuate 3,4-dioxygenase (Uniprot code P20372 and Q8NN15), Catechol 1,2-dioxygenase (Uniprot code P07773 and P95607), Catechol 2,3-dioxygenase (Uniprot code Q53034 and P06622), Gentisate 1,2-dioxygenase (Uniprot code Q0SFK9 and Q13ZY3), Homogentisate 1,2-dioxygenase (Uniprot code Q828S5 and B8H072), and Protocatechuate 4,5-dioxygenase (Uniprot code P20371 and P22635). The gene sequences from the isolates were deposited on European Nucleotide Archive (ENA) and are available at www.ebi.ac.uk/ena/submit/sra/#home.

The short reads were determined by BLAST. All readings with an e-value of less than 0.1 were retained.

#### Determination of Petroleum Hydrocarbons in Soil and in Plant

The procedure followed for hydrocarbons’ quantification in soil was the one described by Guarino et al. (2017). Sieved soil samples were mixed with sodium sulfate (Na_2_SO_4_) to remove excess moisture. Two dichloromethane (CH_2_Cl_2_) in series extraction were stirred (20 ml of CH_2_Cl_2_ for 5 g of soil) in a Turbula for 2 h. The clean-up process was performed in silica gel column. Extracts were concentrated under a nitrogen stream at 35°C for 50 min using a Zymark TurboVap Evaporator and the residue reconstituted a final volume of 10 ml and was analyzed by GC-MS (7890 A, Agilent, United States) according to the method used for the SimDist analysis (Guarino et al., 2017, 2019). Different hydrocarbon fractions were determined (see Table 1).

**TABLE 1:** Concentrations of different hydrocarbons fractions in A, B, C, and D subzone’s soils over time and removal rate of hydrocarbons fractions (T0, T1, T2, T3, TF).

**Subzone**	**Parameter (mg/kg)**	**T_0_ (0 Days)**	**T_1_ (90 Days)**	**T_2_ (150 Days)**	**T_3_ (210 Days)**	**T_*F*_ (270 Days)**	**Removal rate (%) during the different time of treatment**
A	C_1__0__–__1__2_	34±a5	16±b5	10±c2	5±cd2	3±d1	91
	C_1__2__–__1__6_	152±a12	75±b9	44±c10	27±cd12	14±d2	90
	C_16_-C_21_	1292±a121	647±b12	299±c25	151±cd45	73±d12	94
	C_21_-C_40_	2240±a156	1424±b123	1463±b101	885±c85	383±d103	82
	Total C_1__0__–__4__0_	3720±a164	2163±b258	1816±b124	1070±c99	474±d111	87
	TPH	3815±a159	2239±b369	1881±b109	1108±c101	480±d123	87
	HC < 12	38±a10	19±b9	5±c2	4±c2	1±c0.05	84
	HC > 12	3757±a147	2220±b125	1777±b141	1136±c124	489±d103	86
B	C_1__0__–__1__2_	37±a8	18±b5	11±c3	6±cd1	3±d1	91
	C_1__2__–__1__6_	156±a13	79±b8	38±c10	20±cd5	12±d3	92
	C_16_-C_21_	1102±a105	570±b15	289±c28	151±cd14	77±d12	93
	C_21_-C_40_	2335±a153	1576±b132	1577±b56	965±c54	334±d101	85
	Total C_1__0__–__4__0_	3630±a162	2234±b145	1915±b63	1149±c245	424±d120	88
	TPH	3687±a152	2278±b153	1945±b84	1167±c142	429±d118	88
	HC < 12	31±a10	14±b5	3±c1	1±c0.05	1±c0.05	96
	HC > 12	3656±a147	2258±b146	1942±b97	1166±c123	429±d102	88
C	C_1__0__–__1__2_	31±a5	17±b5	9±c1	5±cd1	3±d1	90
	C_1__2__–__1__6_	193±a10	98±b10	50±c5	25±cd10	13±d4	93
	C_16_-C_21_	1037±a123	522±b37	266±c12	138±cd35	70±d10	93
	C_21_-C_40_	2109±a140	1371±b58	1299±b103	667±c45	279±d23	96
	Total C_1__0__–__4__0_	3370±a138	2009±b82	1624±b104	836±c102	365±d25	89
	TPH	3408±a124	2034±b92	1647±b111	846±c113	369±d28	89
	HC < 12	31±a10	14±b5	4±c1	2±c1	2±c1	93
	HC > 12	3378±a123	2021±b104	1644±b95	845±c65	369±d36	89
D	C_1__0__–__1__2_	28±a5	14±b2	4±c2	2±cd0.05	1±d0.03	96
	C_1__2__–__1__6_	183±a11	95±b10	48±c5	25±cd5	13±d4	92
	C_16_-C_21_	987±a45	492±b25	248±c21	126±cd20	57±d15	94
	C_21_-C_40_	1920±a101	1305±b52	1204±b53	619±c62	247±d12	87
	Total C_1__0__–__4__0_	3117±a123	1907±b81	1506±b81	773±c64	319±d15	89
	TPH	3166±a132	1937±b72	1546±b42	780±c33	323±d14	89
	HC < 12	27±a11	11±b3	5±c1	2±c0.02	1±c0.05	96
	HC > 12	3138±a105	1926±b103	1544±b58	780±c13	323±d10	89

*Mean values (n = 3) ± SE followed by non-identical lower-case letters (a-e) indicate significant difference (p < 0.05) among the different times (Duncan’s test).*

For plant analysis, 2 g of each sample were pulverized with sodium sulfate anhydrous (Na_2_SO_4_) by a ceramic mortar and pestle, then extracted and cleaned as described by Guarino et al. (2019) and analyzed by GC-MS (7890 A, Agilent, United States).

#### Analysis of Enzyme in Soil

The polyphenol oxidase (PPO), dehydrogenase (DHO), urease (URE), alkaline phosphatase (ALP), and catalase (CAT) activities were assayed in triplicate air-dried samples as described by Liu et al. (2015).

For DHO activity assessment, 0.03 g of and 0.5 mL CaCO3 of 3% TTC were mixed with a soil sample and incubated at 37°C in the dark for 24 h. After adding 5 mL of methanol, the solution was filtered using a glass funnel capped with absorbent cotton until no red color remained. The samples were diluted to a final volume of 50 mL, adding methanol, and then colorimetrically measured at 485 nm. Control assays (without CaCO3 and TTC) were simultaneously conducted. For URE activity, a solution of toluene, citrate buffer, and urea were mixed with soil and subsequently incubated at 37°C for 24 h. The produced ammonium was determined colorimetrically using the blue Indophenol at 578 nm. A urea-free control was used for each sample. For ALP activity, the soil was mixed with disodium phenylphosphate and borate buffer and incubated at 37°C for 2 h. Potassium hexacyanoferrate in alkaline solution was used to extract and oxidize the phenol produced. Measures were done by the 4-aminoantipyrin colorimetric method at 510 nm. Tests without soil and disodiphenyl phosphate were simultaneously assessed as controls. The catalase activity was spectrophotometrically tested in terms of disappearance of the substrate (hydrogen peroxide). (CAT) activity was tested spectrophotometrically and was measured as nmol of H_2_O_2_ mg^–1^protein min^–1^. An enzymatic unit was calculated as the amount of enzyme that catalyzed the consumption of 1 μmol of H_2_O_2_ per g of medium per hour (Guarino et al., 2018).

#### Assessment of Activity of Stress Marker and Antioxidant Enzyme in Leaves

Fresh leaves (250 g) of each of the four plants were finely ground in liquid nitrogen into a fine powder and were subsequently centrifuged at 19,000 rpm for 30 min. The activity of stress marker and antioxidant enzymes, such as Glutathione S-Transferase (GST), Phenylalanine Ammonia Lyase (PAL), Proline and Lipid peroxidation (MDA), (Superoxide dismutase (SOD), Catalase (CAT), Ascorbate peroxidase (APX), and Guaiacol peroxidase (GPX), were assessed as described by Guarino et al. (2018, 2020).

#### Staining and Microscopy of Mycorrhizal Fungal Colonization

Roots of *Festuca* and *Lotus* species were sampled 150 days after planting to observe the development of host root colonization.

The fresh fine root segments were fixed in FAA (Formalin: acetic acid: ethanol). The fixed roots were then processed with the staining procedures described by Vohník et al. (2016). Mycorrhizal colonization was estimated after clarifying (10% KOH) at 121°C for 15 min, acidifying (% HCl) for 20 s, and staining in 0.05% Trypan blue solution in lactoglycerol (lactic acid/glycerol/deionized water in a mixing ratio of 1:1:3) for 5 min. The excess stain was removed in clear lactoglycerol at room temperature and roots were subsequently placed on glass slides for microscopic observation. The percentage of intracellular hyphal colonization was assessed using a Nikon Eclipse E600 microscope.

### Statistical Data Analysis

The data presented are the mean ± SE. The data were statistically analyzed by means comparison by the parametric ANOVA (considering conservative F statistics for determining significance) and non-parametric Kruskal–Wallis tests (with Bonferroni correction of the *p*-value). Duncan’s *post-hoc* test was used for pairwise comparison. Results of both parametric and non-parametric analyses were quite comparable. Although non-parametric analyses are beneficial because they are free of the assumptions of parametric ones, they are generally considered less robust than parametric analyses (Field, 2009; DePoy and Gitlin, 2016). Hence, only ANOVA analyses results were considered in our study. A one-way ANOVA was adopted to assess whether the hydrocarbons accumulation/translocation (concentration in roots and leaves, respectively) differed among plant species. The two-way ANOVA analyses were performed to test whether the experimental time, plant species, and the interaction between these two factors have an effect on (i) hydrocarbons contamination levels in the pot experiment and (ii) plant antioxidant response (enzymatic activity). A significant difference was considered at the level of *p* < 0.05.

## Results

### Determination of the Physic-Chemical Parameters

The parameters of control soil (CPH) and contaminated soils before (BPH) and after treatment (APH) sites are shown in Supplementary Table S1. It has been observed that the application of NPK fertilizers increased soil nutrients (BPH and APH) with respect to the control soil. In addition, soil with TPH contamination without treatment (BPH) is characterized by a higher nitrogen (1.5 times) and potassium (1–3 times) content as compared to soil after the experimental trial (APH). The lower N levels in APH may be imputed to the uptake of N and P by vegetation, and therefore to their storage function, which could have been enhanced by the AMF activity. In fact, after the treatment, these parameters tend to be very close to those of the control soil. Total organic carbon (TOC) was 1.10 and 0.79% for polluted soil without treatment and uncontaminated soil, respectively. After the treatment, TOC was 0.98%, which is very similar to control sample value.

### Bacterial Diversity and Functional Analyses of TPH Degrading Genes

The three DNA libraries (contaminated soil before and after treatment) were performed. GAIA and KRAKEN analyzed the taxonomic sequenced reads. The reads were assigned to the superkingdom Bacteria for contaminated soil (before and after treatment) (∼95.8%). The dominant phyla were *Proteobacteria* (60%) followed by *Firmicutes* (18.2%), *Actinobacteria* (6.2%), *Bacteroidetes* (6.6%), and *Acidobacteria* (0.5%). In hydrocarbon-contaminated soil, there is an enhancement of *Proteobacteria*, frequently dominated by *Gammaproteobacteria*, which positively reacts to the influx of hydrocarbon substrates and supplemental nutrients supply (Greer et al., 2010; Bell et al., 2014). The presence of *Alphaproteobacteria* was also noticeable (about 30%) with the *Sphingomonadales, Rhospirales, and Rhizobiales* orders. Among the *Betaproteobacteria*, the *Burkholderiales* order was predominant. Within the *Firmicutes*, the *Bacillales* order with the families *Bacillaceae* and *Penibacillaceae* were prevailing; among the *Actinobacteria, Microccaceae* (gen *Arthrobacter*) and *Nocardiaceae* were the uppermost families. Lower percentages of *Bacteroidetes* and *Acidobacteria* were also present. Furthermore, in the context of bacterial diversity, *Chloroflexi, Chlorobi*, and *Nitospirae* were present with modest percentages (1–1.3%). In any case, the soil showed interesting amounts of *Apicoplexa* (protozoa), *Chlorophyta* (algae), and fungi belonging to *Ascomycota* and *Basidiomycota*.

**FIGURE 1:**
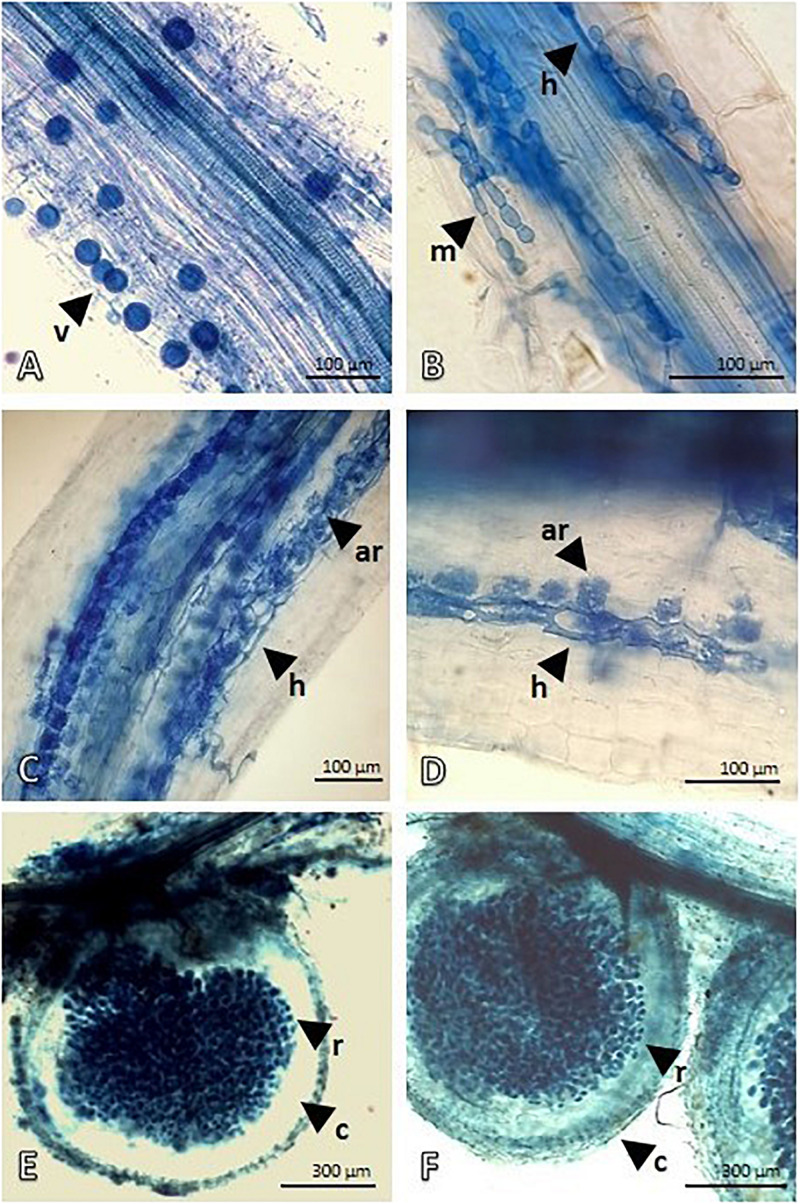
Mycorrhization of *Lotus* and *Festuca* roots and *Rhizobium* nodules of *Lotus* roots detected by Trypan Blue staining and light microscopy. **(A,B)** show the mycorrhize colonization in *Festuca* roots. Intraradical vesicles (v) and hyphae (h) developing intracellular microsclerotia structure (m), with rounded hyphae cell, are visible in root fragments. **(C,D)** show Intraradical hyphae (h) depicting an advanced arbuscule colonization in *Lotus* roots. **(E,F)** display *Lotus* root nodules. Outer, lighter stained, cortex of nodule is well-distinguished from the symbiotic region that is heavily infiltrated by *Rhizobium* bacteria.

After the treatment (APH), the *Proteobacteria* quota increased by about 15% with an increase in *Rhizobiales* and this was attributable to the nitrogen-fixing species introduced (*Medicago* and *Lotus*), but *Pseudomonadales* and *Burkholderiales* also consistently grew. *Firmicutes* with the *Bacillaceae* family also showed a significant increase. However, the panorama of microbial diversity changed in relation to the presence of *Glomeromycota* (microbial consortium introduced) as proof of the mycorrhization success. The presence of *Basidiomycota* and *Ascomycota*, despite being low, was constant. The microscopic analysis (Figure 1) of both *Festuca* and *Lotus* roots clearly demonstrate root colonization, highlighting the presence of typical VAM structures. Figures 1A,B depict *Festuca* root fragments and show intraradical vesicles (v) and hyphae (h). In Figure 1B, an intracellular microsclerotia structure (m) with rounded hyphae cells is also visible. On the other hand, Figures 1C–F represent *Lotus* roots showing an effective nodulation, with nodules heavily infiltrated by *Rhizobium* bacteria. Nodulation evidence demonstrates that our treatment is suitable for nodule formation and contains *Lotus*-compatible rhizobia.

In the context of bacterial diversity, the genera most represented after the treatment were *Pseudomonas*, *Comamonas*, *Achromobacter*, *Acetobacter*, *Sphingobium*, *Burkholderia*, *Bacillus*, *Rhizobium*, and *Bradyrhizobium*, most of which have been recognized as mycorrhizal helper bacteria or root growth stimulators (Poole et al., 2001; Johannsson et al., 2004; Labbé et al., 2011). Obviously, the increase in the fungal component with the *Glomeromycota* has strongly changed the rhizosphere dynamics. Our data also demonstrate that the uniformity of soil bacterial community rises as TPH removal increases, in accordance with previous studies (e.g., Siles and Margesin, 2018). This may be the outcome of a well-established bacterial community in the studied soil, adapted to the existing conditions and to the use of the contaminants present in the system as carbon and energy sources (Serrano et al., 2008). In addition, TPH contents guide the shifts in bacterial community composition.

**FIGURE 2:**
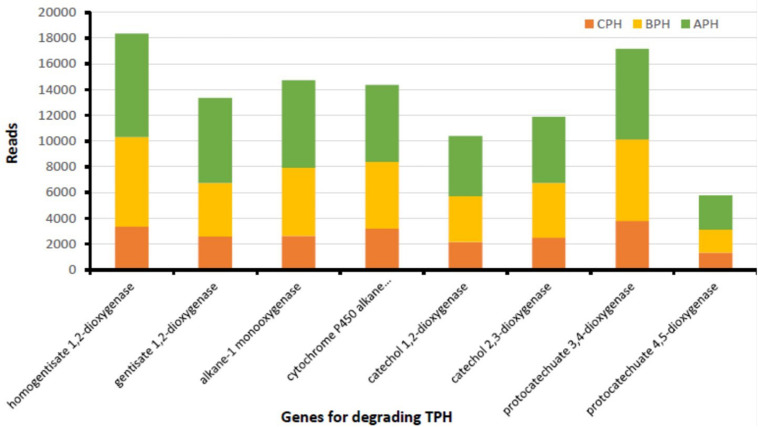
TPH-degrading genes from soil of the contaminated area prior (BPH) and after (APH) the experimental trial and in soil of neighboring uncontaminated area (CPH) used as control.

In soil there is a multitude of enzymes that are important in the conversion of organic substances and energy (Gu et al., 2009). The main class of oxidoreductases are dehydrogenases that are the indicators of global soil microbiological activity (Kaczyñska et al., 2015). Our data demonstrated a high gene abundance of several oxidoreductases involved in hydrocarbon removal, suggesting the degradation prospect of native soil microorganisms. As matter of fact, our data (Figure 2) demonstrate that in hydrocarbon-contaminated soils, both in APH (contaminated soil after treatment) and BPH (contaminated soil before treatment) the degrading genes are far superior than those of CPH (control soil). The APH and BPH soils show abundant amounts of the genes encoding for dehydrogenases, such as naphthalene 1,2-dioxygenase, extradiol dioxygenase, benzoate 1,2 dioxygenase, protocatechuate 4,5-dioxygenase (alpha and beta chain), and 1,2-dihydroxynaphthalene dioxygenase (Figure 2). Therefore, in accordance with our data, it is possible to hypothesize a particular sensitivity of dehydrogenases to the existence of petroleum-related compounds in the soil (as also observed by Kaczyñska et al., 2015). This provides evidence that dehydrogenases have the ability to accelerate the microbiological degradation of such contaminants. We observe the substantial increase in enzymatic activity in correlation with the hydrocarbons’ degradation. These results are in agreement with the current literature, which has shown a strong correlation between the presence of TPH in soils and the abundance of degrading genes produced both by the native micro-channels present and by those introduced as inoculum. In the latter case, co-metabolism factors are triggered, affecting the increase in the enzymatic fitness. In fact, our treatment improved the abundance of hydrocarbon-degrading genes, proving to be suitable for an enhanced soil remediation.

### Determination of the Isolated Hydrocarbon Degrader’s Bacteria in Soil

The *in vitro* tests carried out to show their plant growth promotion potentiality revealed that 72% of the isolated strains are able to produce the phythormones IAA, whereas 66% lead to EPS’s production. In addition, the 78% of isolates exhibit production of siderophores and more than half of them (about 60%) are able to release Ammonia (Supplementary Table S2).

### Degradation of Petroleum Hydrocarbons in Soil

Table 1 shows the TPH levels and their fractions in the four subzones. In all the experiments, the concentrations of hydrocarbon with C < 12 are essentially insignificant (Table 1). Concentrations of hydrocarbon with C > 12 show removal percentages very close to total petroleum hydrocarbon. Statistical analyses revealed that time plays a key role in hydrocarbon concentration levels in soil. The results suggested that grasses’ (Fes and Dac) and legumes’ (Lot and Med) growth increases the removal of TPH from polluted soils. Indeed, cultivated soils had significantly lower hydrocarbons after 90 days with a final TPH removal rate ranging from 87 to 89%.

### Soil Enzyme Activity

Supplementary Figure S2 shows the variation in PPO, DHO, ALP, URE, and CAT activities in rhizosphere soil in relation to days of growth. Rhizosphere soil PPO activity generally rose over time in all subzones with a significant (*p* < 0.05) increase after 90 days (T_1_). It can be associated with the progressive reduction of TPH concentrations in soil. In fact, the correlation between PPO activities of rhizosphere soil (Supplementary Figure S2) and degradation of TPH in soil (Table 1) indicates that soil adapted to TPH pollution by developing polyphenol oxidase after plants growth.

The soil DHO activity did not vary in the different plant species but was affected by TPH degradation amount over diverse growth periods. The DHO activity showed a significant increase after 150 days (T_1_) and continued to rise until the end of the experiment (Supplementary Figure S2).

The soil alkaline phosphatase (ALP) activity significantly enhanced (*p* < 0.05) after 90 (T_1_) and 150 (T_2_) days (Supplementary Figure S2).

The urease activity in rhizosphere soil was largely enhanced after T_1_ (90 days) and T_2_ (150 days). These results denote that the presence of hydrocarbons in the soil did not discourage URE activity at the initial stage of plant growth, suggesting the existence of a stabilized portion of urease that is extremely resistant to pollutants. Results also show that soil URE activity of the four species continuously rose over time (Supplementary Figure S2). The rise of URE activity is helpful for plants because it supports the hydrolysis of nitrogen and carbon in contaminated soil, facilitating the degradation hydrocarbons in soil.

The dependence of catalase (CAT) soil activity over time showed an increasing trend. Statistical differences were observed after 90 and 150 days. CAT is another intracellular enzyme developed by all aerobic bacteria and most facultative anaerobes, and is an indicator of the soil community activity and soil fertility (Woliñska et al., 2016).

We share the opinion that increased enzymatic activity of soil is a footprint of the highly consolidated associations between plants and microorganisms, both symbiotic and plant growth-promoting rhizobacteria (Gianfreda, 2015). This interaction also improves the transformation and/or degradation of polluting compounds. Therefore, our data shows that soil enzymatic activities were more substantial after 90 days (T_1_) and enhanced at the end of the experiment (270 days), a tendency that is correlated to the amount of TPH degradation rate in soil.

### Petroleum Hydrocarbons in Plants

**FIGURE 3:**
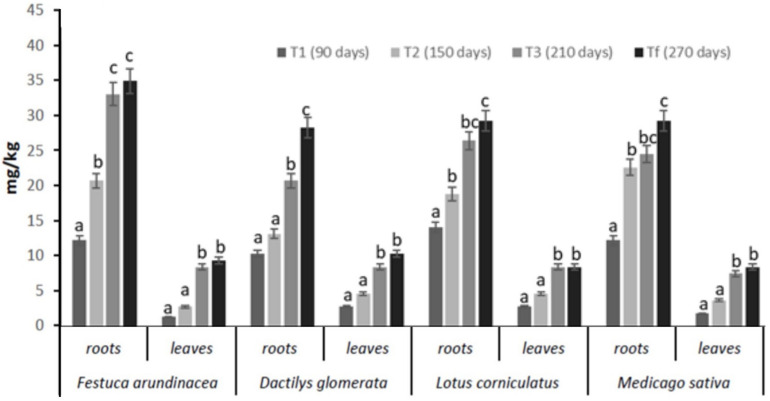
Levels of TPH in roots and leaves of *Festuca arundinacea*, *Dactylis glomerata*, *Medicago sativa*, and *Lotus corniculatus* after 90 (T_1_), 150 (T_2_), 210 (T_3_), and 270 (T_*f*_). Means ± SE with different letter indicate significant difference according to Duncan’s test (*p* < 0.05).

The concentrations of total petroleum hydrocarbons (TPH) found in plant samples (roots and leaves) are shown in Figure 3. Our results indicated that *Festuca arundinacea*, *Dactilys glomerata, Lotus corniculatus*, and *Medicago sativa* have good remediation potential in TPH-polluted soils. Statistical analyses revealed that a significant difference between *Festuca* and the other plant species was found in terms of overall hydrocarbons root concentrations. In fact, TPH concentrations in *Festuca* samples ranged between 13 and 38 mg/kg in roots and 1.5–10 mg/kg in leaves from 90 days (T_1_) to 270 days (T_*f*_). In *Dactilys*, TPH concentrations in samples ranged between 14 and 30 mg/kg in roots and 5–11 mg/kg in leaves from 90 days to 270 days.

In *Lotus*, TPH concentrations were between 15 and 31 mg/kg in roots and 3–9 mg/kg in leaves from 90 days to 270 days. In *Medicago*, TPH concentrations in samples ranged between 13 and 31 mg/kg in roots and 3–9 mg/kg in leaves from 90 days to 270 days. These results showed that TPH concentrations in plant samples were higher in roots and lower in leaves.

Comparing the TPH content in both analyzed parts (roots and leaves) of the plant, a higher accumulation of TPH through the roots occurs. These results indicate that the uptake of petroleum hydrocarbons in roots is enhanced through a rhizodegradation mechanism. In rhizodegradation, organic pollutants are degraded in soil through the bioactivity of the rhizosphere, which derives from the production and exudation of enzymes and proteins by plants or soil microorganisms such as bacteria or fungi (Guarino et al., 2020). The lower levels of TPH in leaves may be related to the hydrocarbons phytotransformation or phytodegradation occurring within the plant itself.

### Stress Marker and Antioxidant Enzyme Activity in Plants

Reactive oxygen species (ROS) are generated in several redox reactions in plants since they play the role of signaling molecules involved in various phases (e.g., growth and development). However, environmental stress, such as pollution of soil in which plants grow, can favor the intracellular levels of ROS due to the alteration of the equilibrium between ROS production and scavenging (Hayat et al., 2012). To avoid cellular damage caused by oxidative stress, plants accumulate osmolytes (e.g., proline, glycine-betaine, polyamines, and sugars) to safeguard the cellular machinery and inhibit the production of harmful ROS. In our experiment, antioxidant enzymes and stress marker activities were determined in leaves of the four plant species (Supplementary Figure S3) to evaluate the defense strategy set under abiotic stress (such as TPH pollution) to regulate ROS toxic levels and avoid oxidative damage. Statistical analyses highlight that the variations in the activities of all the evaluated enzymes were related to the progression of the experimental time. The activity of SOD in all studied species decreased significantly (*p* < 0.05) over experimental time. The enhanced SOD activity in leaves at T1 (90 days) could be related to a higher TPH exposure, even in the presence of microorganisms able to promote plant growth. CAT activity in the leaves of both Poaceae and Fabaceae showed decreasing patterns similar to SOD activity, lowering over time. GPX and APX pattern unveiled that a significant reduction is observed over longer periods of time.

In order to estimate the level of oxidative stress, the malandialdehyde content (MDA), Glutathione S-Transferase (GST), Phenylalanine Ammonia Lyase (PAL), and Proline content were determined, and our results indicate a decreasing pattern over the experimental time. MDA is a widely used marker of oxidative lipid damage caused by enhanced levels of ROS under stress. After 270 days, MDA level decreased in leaf samples of all the investigated plant species. These findings are in accordance with other research highlighting that MDA levels in leaves increased with increasing crude oil concentration in soil and decreased with exposure time (Shukry et al., 2013; Guarino et al., 2020). The decreasing MDA trend over time may indicate that, under hydrocarbons’ degradation, repairing mechanisms start to keep pace with oxidative damage. As regards proline, at an earlier time we observed higher contents, which decreased as TPH concentration diminished in the soil. Our data may suggest that the investigated plants suffer stress at early stages due to the high levels of soil contamination and thereafter respond by enhancing their tolerance and inhibiting the production of harmful ROS.

## Discussion

For effective phytoremediation, it is mandatory to gather key information from field experiments. A theoretical approach based only on laboratory data may not be sufficient. Unfortunately, the geo-environmental conditions are site-specific, and therefore bioremediation models must be contextualized to the actual location where reclamation will take place. Using integrated remediation systems is very useful, especially when pollutants are hydrocarbons. In fact, the choice to use landfarming first and then biostimulation is based on two main objectives: (1) to obtain a quota of degradation through thermal (solar) and oxygenation processes and (2) to create an environment more conducive to the microbial growth of the potential autochthonous microflora present on site. Also. the identification of particular microorganisms in soils to be reclaimed that have both a degradation activity and/or act as Plant Growth-Promoting Rhizobacteria (PGPR) is essential for the success of the intervention. In our case, 33 bacteria were isolated, showing different and marked activities such as production of IAA (Indole Acetic Acid), siderophores capacity, or production of EPSs (Extracellular Polymeric Substances).

The significance of nutrients in hydrocarbon-contaminated soils has long been recognized since they support microbial activities. Macronutrients, such as N-P-K, are also essential for the production of amino acids and the transport of energy (such as adenosine triphosphate, which is needed for symbiotic N-fixation). In fact, many authors have described that soil fertilization with NPK fertilizer results in enhanced TPH rhizoremediation up to 65% (Vaajasaari et al., 2006). Hence, nutrient external supplements to enhance the development of microbial flora and improve plant growth has been considered to promote the hydrocarbons’ removal. The plant species chosen in this study for hydrocarbons’ phytoremediation are characterized by suitable features, such as (i) a remarkable tolerance to specific contaminants, (ii) extensive root systems, and (iii) a marked susceptibility to AMF (Arbuscular Mycorrhizal Fungi) infections. In particular, Poaceae have a fibrous root system (with a high specific surface) and are able to actively explore deeper soil layers (up to 100 cm), which improves the contact between contaminants and degrading microbes (Cougnon et al., 2017). In addition, the chosen Poaceae can emit high quantities of soluble organic substances and secondary metabolites in the rhizosphere, which are directly and indirectly involved in the degradation of pollutants. The root infection with mycorrhizal fungi was also a goal of our experiment since it is well-recognized that mycorrhization can (i) enhance the hydrocarbon degradation in soils, (ii) improve nitrogen fixation (especially for Fabaceae), which is of particular interest for its ecological and agronomic implications (Hernández-Ortega et al., 2012), and (iii) alleviate hydrocarbon toxicity on plants.

Many authors have highlighted the role of Fabaceae in phytoremediation as these taxa offer wide perspectives of adaptations to soils contaminated by oil (Hall et al., 2011). The symbiotic association between rhizobia and Fabaceae may result in N fixation and thus in soil fertility and productivity improvement. Therefore, the plant species chosen in this field experiment have all the characteristics of suitability for soil function rehabilitation. *Medicago sativa* has an extensive and efficient root system both in terms of production of compounds (exudates) very useful for the native microbial flora and for the ability to develop symbiosis with nitrogen-fixing bacteria. *Lotus corniculatus* is among the most frequent species in disturbed areas of the Mediterranean basin, a rustic and N-fixing species. Both *Festuca* and *Dactylis* represent pioneer plants of nutrient-poor soils and polluted soils and have been used in various field and laboratory experiments due to their efficiency in TPH degradation. In our in-field experiment, *Festuca arundinacea* (Poaceaer family) with inoculation of endophytic fungi showed more root biomass and enhanced hydrocarbons’ root accumulation and degradation.

**FIGURE 4:**
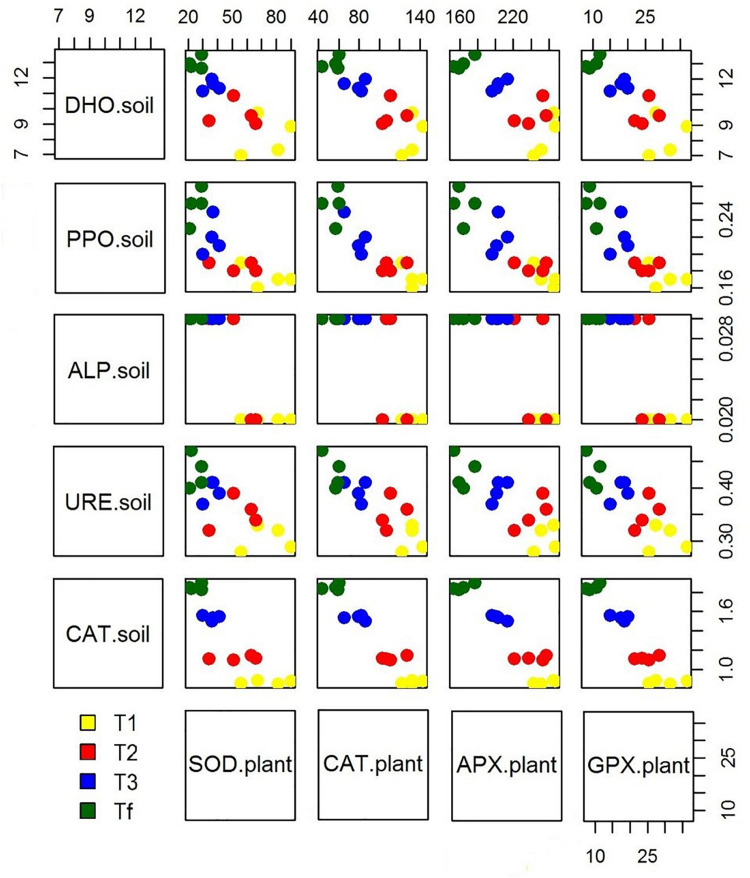
Scatterplot matrix showing the relationship between the soil enzymatic activity and plant stress markers over experimental trial time [0 days (T_0_), 90 days (T_1_), 150 days (T_2_), 210 days (T_3_), and 270 days (T_*f*_)].

Because of hydrocarbon hydrophobicity, there could have been problems for plants in the assimilation of water and nutrients. However, many studies have shown that these issues can be overcome by introducing mycorrhizae. Furthermore, the beneficial effects of mycorrhizae on the growing process of plants exposed to abiotic stress is reflected in the ability to obtain a new homeostatic equilibrium with the regulation of stress markers. The microorganism-plant associations improve the transformation and/or degradation of polluting compounds, ultimately resulting in their removal and thereby in lower level of abiotic stress for plants. As a matter of fact, our data showed that as soil enzymatic activity grows over time, a reduced antioxidant response in plants can be observed (Figure 4). Our observations are in accordance with other studies (e.g., Balseiro-Romero et al., 2017; Rusin et al., 2018) which observed that plant-microbial interactions could decrease antioxidant enzymes activity in plant growing in diesel-contaminated soil. The significant reduction of enzyme activities in plants may reveal an enhanced abiotic stress tolerance, which is directly related to the growing dissipation of hydrocarbons and indirectly linked to the rhizosphere effect. Moreover, the degradation of hydrophobic petroleum compounds may have improved the water holding capacity in soils (Hussain et al., 2019), resulting in increased available water content for plants. In addition, the co-activity of plants and associated microorganisms enhances nutrient availability in soil (Gkorezis et al., 2016), with a direct effect on the biofortification of plants.

The degradation of hydrocarbons in our site may be mainly based on AMF-mediated rhizodegradation. The mycelium produced by mycorrhizae fungus increases the rhizosphere surface allowing a greater root exploration, forming the hyphosphere which represents a favorable habitat for soil microorganism activity and proliferation (Andrade et al., 1997).

In rhizodegradation, both the enzymes of the microbiota and those produced by the roots contribute to the progress of hydrocarbon degradation. Monitoring the enzymatic activities at the rhizosphere level is essential to understand the evolution of the degradation process and the success of the experiment. In the structure of the hyphosphere, the high activity of oxidoreductase, such as dehydrogenase, catalase, polyphenol oxidase, peroxidase, dioxygenase and superoxide dismutase, was observed. In our case, the enzymatic activities found in rhizosphere soil (PPO, DHO, ALP, and URE) increase proportionally with the development of rhizosphere biomass and the degree of infection of AMF. In fact, the more this symbiotic relationship is consistent, the more it will increase rhizosphere biomass and the consequent enzymatic activities.

The integrated biological systems of phytoremediation exploit the synergistic effect of the plant-bacteria-mycorrhizae relationship. In this context, bacteria can act as “mycorrhizae helpers.” Some endophytic PGPR that release cellulose and pectinase (Yu et al., 2011) can also facilitate the penetration of the root by fungal hyphae. This observation was also found in our experiment due to the presence of different bacterial strains with marked qualities of PGPR. A small plant root section (1 cm) can be colonized by many fungal species with different mutual benefits. The occurrence of the mycorrhizo-rhizobic associations is very evident in the root apparatuses of Fabaceae used in our experiment.

Although the common paradigm is that plants are not able to uptake hydrocarbons from the soil, our findings suggested that plants themselves may play a role in taking up a fraction (even small) of TPH into their tissues, in accordance with the evidence provided by many studies (Hunt et al., 2019 and references therein). Our experiment showed that hydrocarbon translocation into the aerial part of plants is limited and fixation occurs mainly in roots (Zhang et al., 2017), mainly due to the high molecular mass and hydrophobic properties. In our experiments the mycorrhizae colonization may have promoted the accumulation of hydrocarbons in plants roots, although this pathway represented only a partial fraction of their overall removal from soil. It is noteworthy that the conformation and the structure of hydrocarbons is a factor determining the success of the experiment. In our study, the presence of heavier hydrocarbons (C > 12) strongly influenced the degradation rate of TPH. Indeed, in all the four subzones, the degradation chrono-sequence revealed that hydrocarbons with shorter chains (C < 12) are better degraded than the more recalcitrant ones which may require a longer time.

## Conclusion

The integrated biological systems used for the reduction of hydrocarbons in soils are an interesting form of technology with great prospects, which may result in improving environmental quality, protecting human health, and restoring agricultural soil functions through relatively low-cost protocols. In order for these systems to achieve positive effects on bioremediation, the right combination of different biological elements and good agronomic practice must be well-designed. First, preliminary site investigations, to acquire an understanding of all aspects involved, are fundamental for the success of the work. Moreover, the chronosequence of the various operations is necessary for the final effectiveness. Therefore, land-farming-biostimulation-phytoremediation with assisted HMF are phases of a single process which, with the progress of the work, increases its effectiveness more and more, since it is an extremely efficient system in adapting to the site conditions.

The AMF-assisted land-farming-biostimulation-phytoremediation design should include (i) the right combination of nutrients and (ii) the best plant-microorganism combination, which may enhance ecotype resistance to toxic organic pollutants. Special consideration should be given to the features of the plant root system, which should meet the requirements of having (i) a rapid and maximum extension, (ii) considerable biomass renewal, (iii) susceptibility to AMF infections, and (iv) a high rate of root exudates release, which represent a source of hydrophobic petroleum compounds degrading enzymes and easily available C.

The extension of rhizosphere capacity through the hyphae networks produced by mycorrhizae results in the development of a native hydrocarbon-degrading microbial flora and/or PGPR that participate directly or indirectly in the degradation and extraction of hydrocarbons. The monitoring activities that we propose in our biological system are very effective in understanding the synergistic and progressive mechanism that is established at the level of rhizosphere soil. These multiple and progressive biological systems allow us to address bioremediation problems which had previously been unsolvable due to the lack of knowledge. The importance of real-scale experimentation is fundamental for a correct technological transfer of the combinations adopted. This is the only way to understand the real mechanisms of symbiotic aggregation, of plant homeostatic response, and consequent activity of the rhizosphere complex. Our full-scale experiment has actually produced positive effects on the degradation of TPH and provided a wealth of knowledge on the dynamics of the various synergistic activities (PGPR, rhizosphere enzymes, etc.) existing in the soil environment under abiotic stress conditions. The signaling path between plants and associated microorganisms would be a promising line of research for future studies aimed at improving the rehabilitation of industrial soil and offering more sustainable management in agriculture conditions.

## Data Availability Statement

The datasets generated for this study can be found in the online repositories. The names of the repository/repositories and accession number(s) can be found below: https://www.ebi.ac.uk/ena/submit/sra/#home.

## Author Contributions

DZ, CG, MT, and RS contributed to conception and design of the study. RS organized the database. DZ analyzed the data and wrote the original draft of the manuscript. CG supervised. All authors contributed to manuscript critical evaluation, revision, read, and approved the submitted version.

## Conflict of Interest

The authors declare that the research was conducted in the absence of any commercial or financial relationships that could be construed as a potential conflict of interest.

## Footnotes

1 www.ncbi.nlm.nih.gov/blast/Blast.cgi
