# Supplementary material for: Plant-Soil-Microbiota Combination for the Removal of Total Petroleum Hydrocarbons (TPH): An In-Field Experiment
Source: Front Microbiol. 2021 Jan 26;11:621581. doi: 10.3389/fmicb.2020.621581 (PMC7873869; doi:10.3389/fmicb.2020.621581)

**Supplementary Materials for**

**Identification of the best plant-soil-microbiota combination for the removal of Total Petroleum Hydrocarbons (TPH): an in-field experiment**

**Daniela Zuzolo^1^, Carmine Guarino^1*^, Maria Tartaglia^1^, Rosaria Sciarrillo^1^**^.^

University of Sannio-Department of Science and Technology, via De Sanctis snc, 82100 Benevento, Italy

*** Correspondence:**  [guarino@unisannio.it](mailto:guarino@unisannio.it)

Table S1 – Average physico-chemical properties of polluted soil before treatment (BPH), polluted soil after treatment (APH) and control soil (CPH).

| **Parameter** | **Control soil (CPH)** | **Polluted soil before treatment (BPH)** | **Polluted soil after treatment (APH)** |
| --- | --- | --- | --- |
| Texture of soil | Fine loamy soil | Fine loamy soil | Fine loamy soil |
| Temperature (°C) | 37 | 37 | 37 |
| pH | 8.26 | 8.10 | 8.12 |
| Organic carbon (%) | 0.79 | 1.10 | 0.98 |
| Total Nitrogen (Kgha^−1^) | 1373 | 2079 | 1456 |
| Available P_2_O_5_ (Kg ha^−1^) | 25.63 | 38.12 | 32.11 |
| Available K_2_O (Kg ha^−1^) | 123 | 175 | 144 |
| EC (dSm^−1^) | 0.18 | 0.34 | 0.22 |

Table S2 - Primary screening of potential PGPR activities by bacteria isolates recovered from BPH rhizosphere soil. The isolates were categorized into three groups according to the produced amount: + low concentrations (<1 µg/ml), ++ moderate concentrations (1 -2.99 µg/ml) and +++ high concentrations (>3 µg/ml).

| Bacterial isolates | Production IAA | Siderophore release | Exopolysaccharides (EPSs) production | Production of ammonia |
| --- | --- | --- | --- | --- |
| *Gordonia polyisoprenivorans* VH2 | +++ | +++ | +++ | +++ |
| *Achromobacter marplatensis* | - | - | - | - |
| *Bacillus thuringiensis* | +++ | +++ | ++ | + |
| *Bacillus spp.* | +++ | +++ | ++ | + |
| *Bacillus licheniformis* | +++ | +++ | ++ | + |
| *Achromobacter xylosoxidans* | ++ | - | - | ++ |
| *Comamonas spanius* | +++ | +++ | +++ | +++ |
| *Comamonas testosteroni* | ++ | ++ | ++ | ++ |
| *Ochrobactrum anthropi* | +++ | +++ | +++ | +++ |
| *Pseudomonas* *migulae* | ++ | ++ | + | + |
| *Pseudomonas aeruginosa B136* | ++ | ++ | ++ | + |
| *Pseudomonas* *chlororaphis* | ++ | ++ | + | + |
| *Pseudomonas mandelii* | + | - | + | - |
| *Pseudomonas resinovorans NBRC 106553* | ++ | ++ | +++ | +++ |
| *Pseudomonas fluorescens F113* | ++ | ++ | +++ | ++ |
| *Pseudomonas putida H8234* | +++ | +++ | ++ | ++ |
| *Pseudomonas stuzeri JBC1* | +++ | +++ | ++ | ++ |
| *Pseudomonas brassicacearum NFM421* | ++ | +++ | + | ++ |
| *Pseudomonas corrugata* | ++ | + | + | + |
| *Pseudomonas koreensis* | - | ++ | - | - |
| *Pseudomonas plecoglossicida* | + | - | + | + |
| *Pseudomonas alcaliphila* | - | ++ | - | - |
| *Sphingomonas sanxanigenens NXO2* | - | - | - | - |
| *Sphingobium chlorophenolicum* | ++ | ++ | - | - |
| *Burkholdera* c*epacia GG4* | ++ | ++ | ++ | ++ |
| *Burkholderia pseudomallei NTC 13179* | *++* | ++ | ++ | ++ |
| *Rhodococcus pyridinivorans* | - | ++ | - | - |
| *Rhodococcus sphaeroides* | - | +++ | - | - |
| *Paenibacillus mucilaginosus 3016* | ++ | ++ | - | - |
| *Paenibacillus polymyxa* | +++ | - | - | - |
| *Sphingobium abikonense* | ++ | + | ++ | + |
| *Bradyrhizobium japonicum USDA6* | - | ++ | ++ | - |
| *Bradyrhizobium diazoefficiens* | - | + | + | - |

Figure S1 – Study area. The perimeter of refinery is shown in green; yellow polyline depict the experimental area (2000 m^2^); the four red points represent the center of the four subzones (A, B, C, D). The black dots indicate soil sampling locations (n=5) for each subzone.


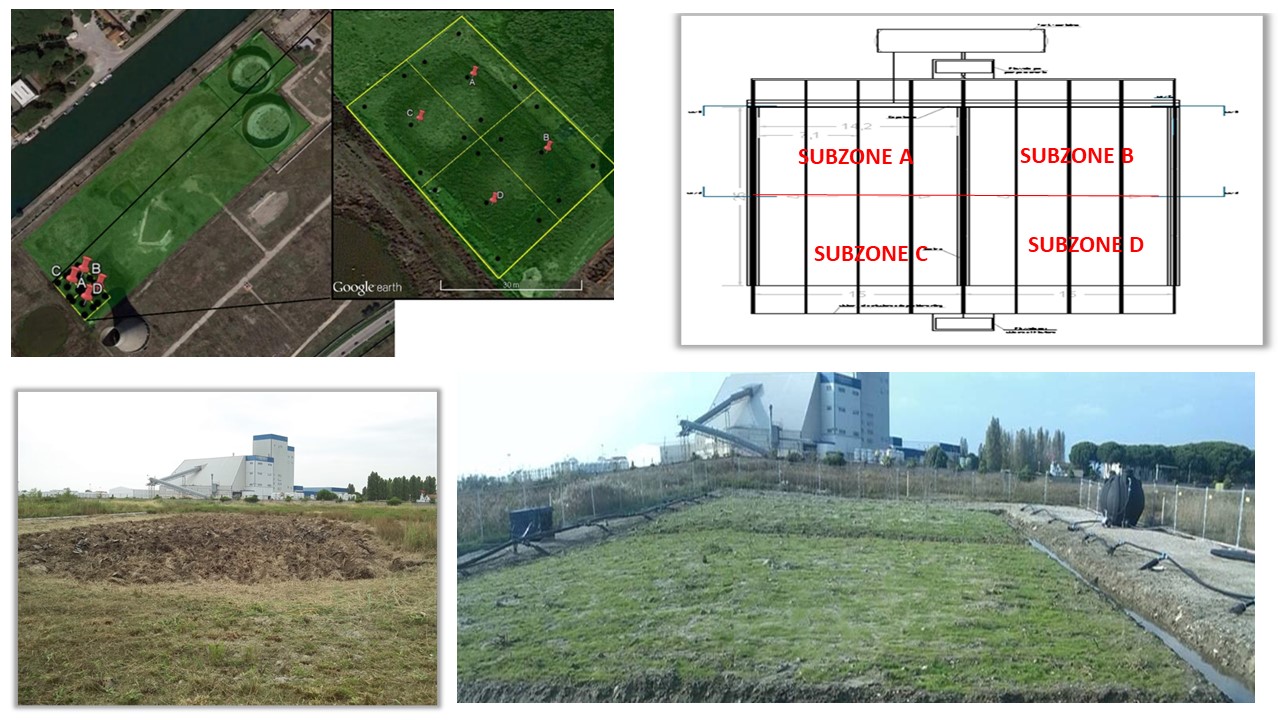


Figure S2 - The polyphenol oxidase (PPO) activity, the dehydrogenase (DHO) activity, the urease (URE) activity and the alkaline phosphatase (ALP) activity, the catalase (CAT) activity in soil of four subzones (A, B, C and D) at different times: days 0 (T0), 90 (T1), 150 (T2) 210 (T3) and 270 (Tf). Means ± SE with different letter indicate significant difference according to Duncan's test (p <0.05).


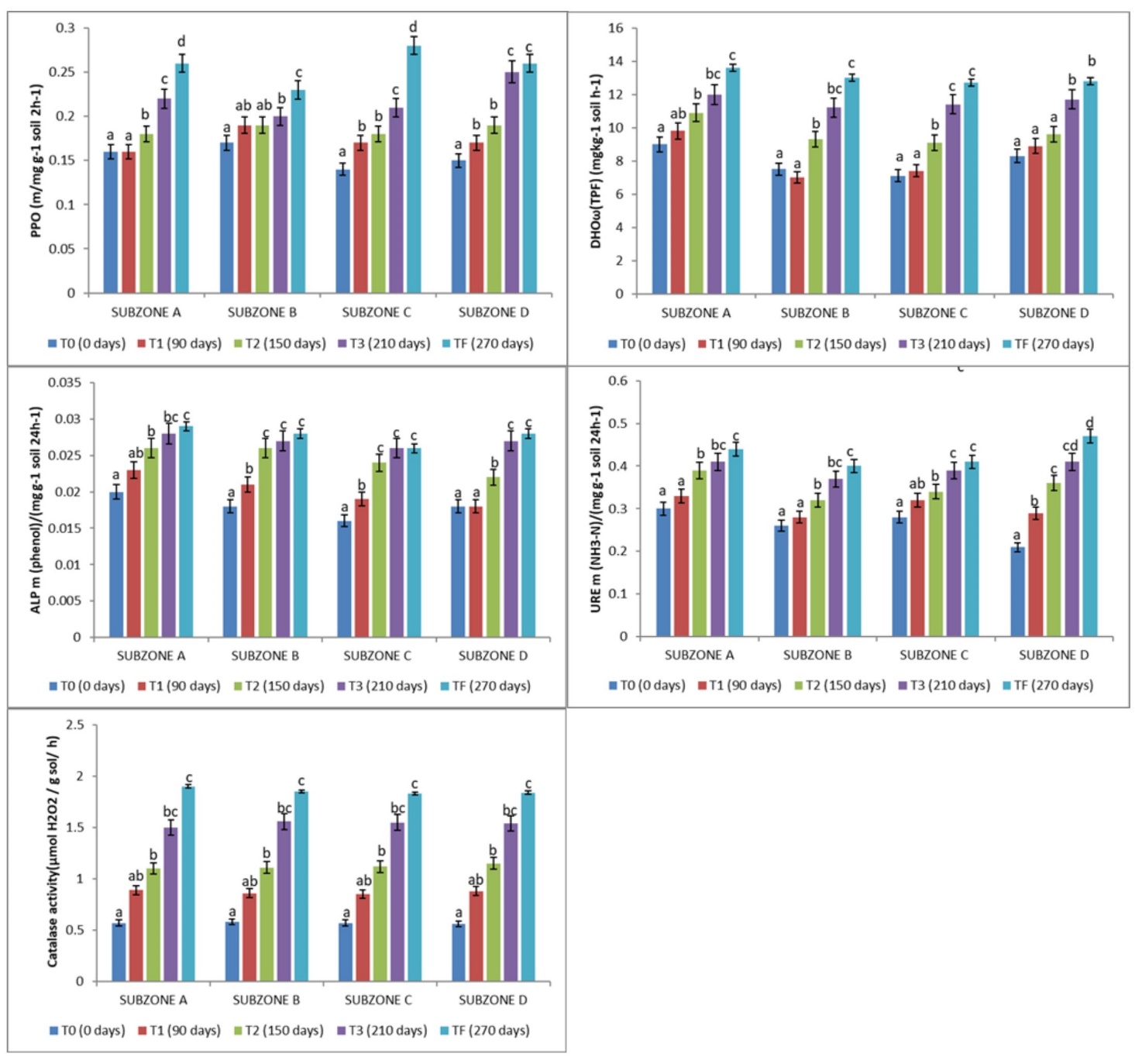


Figure S3 - Activity of SOD, CAT, GPX and APX, GST, PAL, Proline content and MDA in leaves of Festuca arundinacea (Fes), Dactylis glomerata (Dac), Medicago sativa (Med) and Lotus corniculatus (Lot) after 90 ( T1), 150 (T2) 210 (T3) and 270 (Tf). Means ± SE with different letter indicate significant difference according to Duncan's test (p <0.05).


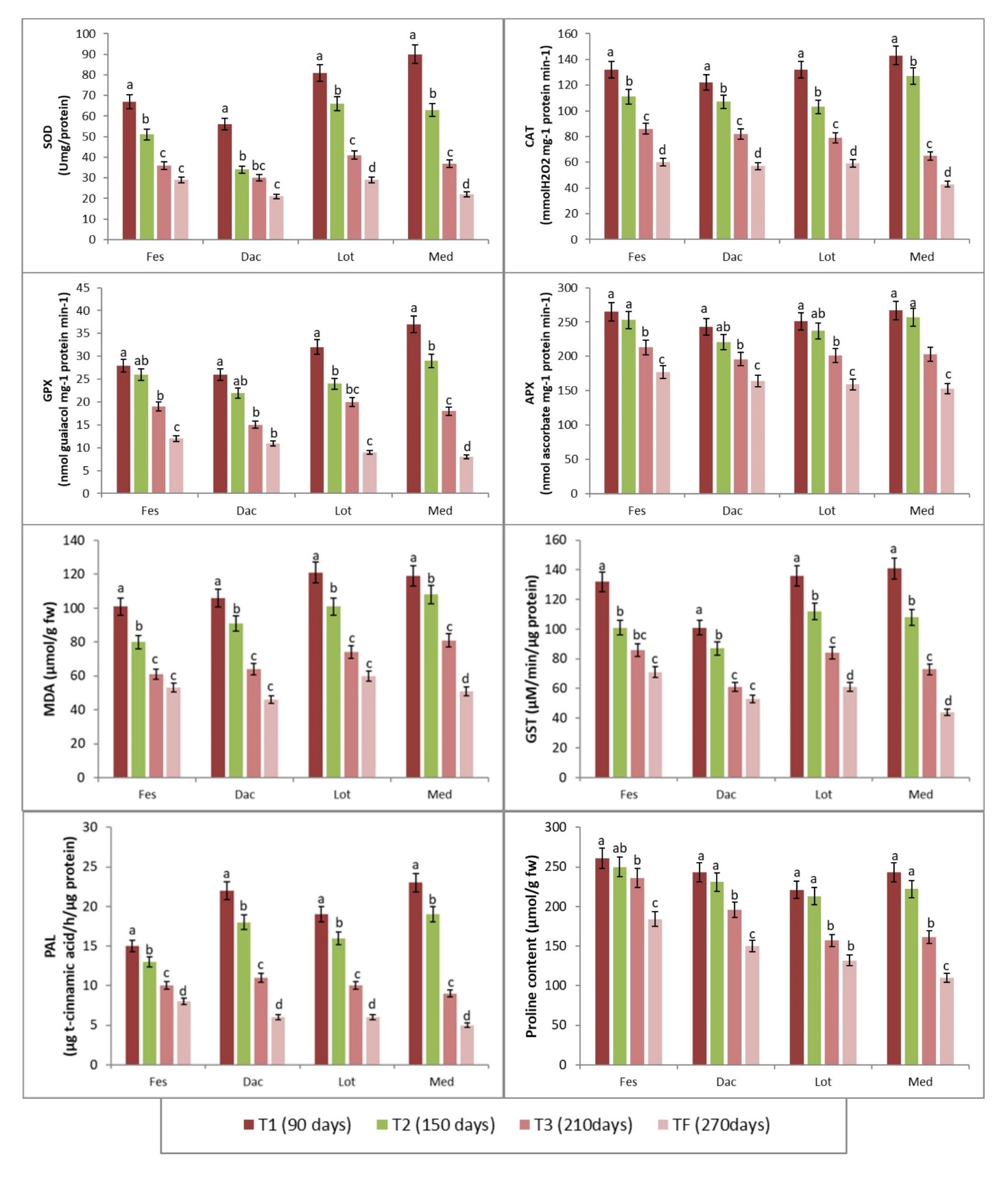

Supplement: Supplementary file 1 [file Data_Sheet_1.docx]
